# Physiotherapists’ Adoption of a Theory-Based Skills Training Program in Guiding People with Rheumatoid Arthritis to Health-Enhancing Physical Activity

**DOI:** 10.1007/s12529-018-9716-1

**Published:** 2018-07-03

**Authors:** Thomas Nessen, Christina H. Opava, Ingrid Demmelmaier

**Affiliations:** 1 Department of Neurobiology, Care Sciences and Society, Division of Physiotherapy, Karolinska Institutet, 23100, SE14183 Huddinge, Sweden; 2 Department of Rheumatology, Karolinska University Hospital, Stockholm, Sweden

**Keywords:** Adherence, Behavior change techniques, Education, Fear-avoidance beliefs, Intervention, Physical activity, Physical therapist, Self-efficacy

## Abstract

**Purpose:**

To describe physiotherapists’ (PTs’) adoption of a theory-based skills training program preparing them to guide people with rheumatoid arthritis (RA) to health-enhancing physical activity (HEPA) within a 1-year intervention trial.

**Method:**

This was a longitudinal case study. Ten female PTs (age 25–59), delivering the HEPA intervention, participated. Data were collected on five occasions over a 19-month period: once before the training course, once after 4 course days, twice during the HEPA intervention and once after the HEPA intervention. Knowledge on about physical activity (score 0–6) and behavior change techniques (BCTs) (score 0–18), fear-avoidance beliefs (score 8–48) and self-efficacy to guide behavior change (score 9–54) were assessed with a questionnaire. Structured logbooks were used to register PTs’ self-reported guiding behavior. Criteria for PTs’ adherence to the protocol were pre-set.

**Results:**

PTs’ knowledge on about BCTs and their self-efficacy increased significantly (*p* < 0.05) from median 9 to 13 and from median 38 to 46.5, respectively. Knowledge on about physical activity was high and fear-avoidance beliefs were low before the education (median 6 and 13.5, respectively) and did not change over time. Two out of ten PTs fulfilled the pre-set criteria for adherence throughout the intervention.

**Conclusion:**

The results suggest that a theory-based skills training program improves PTs’ knowledge on about behavior change techniques and their self-efficacy to guide people with RA to HEPA. PTs’ adherence to the protocol was not complete but the clinical relevance of the adherence criteria need to be validated against observed PT behavior and patient outcomes.

## Introduction

Physical therapists (PTs) are raised in a strong biomedical tradition focusing on physical aspects of functioning when assessing and treating patients [1]. However, when it comes to guiding patients to improved health behaviors such as health-enhancing physical activity (HEPA) [2, 3], PTs need a wider approach combining their established biomedical knowledge on exercise and physiology with skills to guide behavior changes in patients. Such an approach includes specification of physical activity, not only by type, frequency, duration, and intensity, but as a number of specific behaviors with physical, psychological and contextual determinants [4]. It also includes use of evidence-based behavior change techniques (BCTs) [5] such as specific goal setting, action planning and self-monitoring of physical activity [6, 7]. Previous research has indicated that integration of biomedical and behavioral knowledge and skills in clinical settings may be challenging to PTs [8–11].

Health professionals’ adoption of new approaches and changes in clinical behavior can be facilitated by education, with active and targeted interventions proving more effective than passive methods focusing only on increasing knowledge [12, 13]. Clinical behaviors may be influenced by health professionals’ beliefs about outcome, e.g. fear-avoidance beliefs related to patients’ physical activity [14]. Hence, health professionals with strong fear-avoidance beliefs are more likely to advise patients with back pain to limit their activities, despite clinical guidelines recommending advice to stay active [15]. Another belief predicting behavior is self-efficacy, defined as perceived own capability to perform a specific behavior in order to reach a goal [16]. It is a generic, strong determinant of individual behavior [17], including clinical practice among health professionals [18, 19].

Rheumatoid arthritis (RA) is a long-term autoimmune disease affecting 0.5–1% of the population and is characterized by inflammation, pain, swollen joints and cardiovascular comorbidity [20]. Besides medication, physical activity is highly recommended as treatment [20, 21]. Recommendations on HEPA for people with RA are mainly the same as for the general population: at least 150 min per week of moderately intense aerobic physical activity, combined with twice-weekly muscle strength training [2, 3]. Despite the beneficial effects of physical activity, most people with RA do not perform sustained HEPA [22, 23]. PTs play a central role in promoting HEPA among patients with RA, but their ability to integrate a biomedical approach with that of behavior change guidance when doing so has not been investigated.

The Physical Activity in Rheumatoid Arthritis (PARA) 2010 study evaluated a novel 1-year HEPA intervention, integrating aerobic moderate-intensity physical activity such as walking or biking, circuit training at public gyms and guidance of study participants in behavior change [24]. PTs were trained to guide participants in a skills training program based on social cognitive theory [25] targeting physical activity and behavior change. The study result indicated an increase in physical activity behavior among the study participants [26]. Thus, a combined biomedical and behavioral approach on HEPA promotion seems feasible; however, we need more knowledge on how PTs can adopt such an approach.

The present study aimed to investigate, within a 1-year HEPA intervention trial, PTs’ adoption of a theory-based skills training program in guiding people with RA to increase their physical activity towards HEPA. More specifically, we aimed to assess any changes in PTs’ knowledge, their fear-avoidance beliefs on physical activity in RA, and their self-efficacy to guide behavior change, as well as their self-reported behavior in terms of adherence to a structured protocol when guiding people with RA to HEPA.

## Methods

### Design

This longitudinal case study was performed within the context of a 1-year HEPA intervention conducted in six Swedish cities. It followed a process of change allowing a close-up description of a small number of cases in a defined setting [27].

#### The HEPA Intervention

The 220 HEPA intervention participants were to perform 150 weekly minutes of moderate-intensity aerobic activity (e.g. walking and biking) and twice-weekly circuit training sessions at public gyms. In addition, they were to attend biweekly PT-led support group sessions (with 5–14 people, average 9.6) to facilitate HEPA. These sessions were held in conference rooms adjacent to the gyms. Study-specific participant handbooks and PT manuals were used for all 20 sessions over a year. The handbooks and manuals included information on HEPA and on specific themes such as pain, strength training, sleep and stress, each of which was addressed at one session. Information on BCTs was also provided along with tools to facilitate their usage. Additional information on the HEPA intervention is published elsewhere [24].

### Study Sample

Twelve PTs were enrolled in the study. One was excluded as she was part of the PARA 2010 research team, while another was excluded as she resigned from the study for personal reasons halfway through the intervention. All ten participants finally included in the analyses were female registered PTs, between 25 and 59 years old (median 42.5), with 2–32 years (median 15) of professional experience. Nine had experience within rheumatology (maximum 22 years, median 6).

Eight PTs had previous education in behavioral science and methods. This included motivational interviewing (*n* = 7, with 2–8 days of introductory training), cognitive behavioral therapy (*n* = 3, between 5 and 20 weeks of education), and university studies in psychology (*n* = 4, between 5 and 20 weeks of education). Seven PTs had previous continuing education on physical activity in RA; of these, four had university training on the subject (between 5 and 20 weeks). The PTs were recruited from six rheumatology clinics in Sweden, volunteering to participate in the HEPA intervention study. Each clinic had one or two PTs employed as support group leaders in the study. At one of the sites, one additional PT was recruited from a cardiovascular disease rehabilitation clinic.

### The PT Skills Training Program

**Fig. 1:**
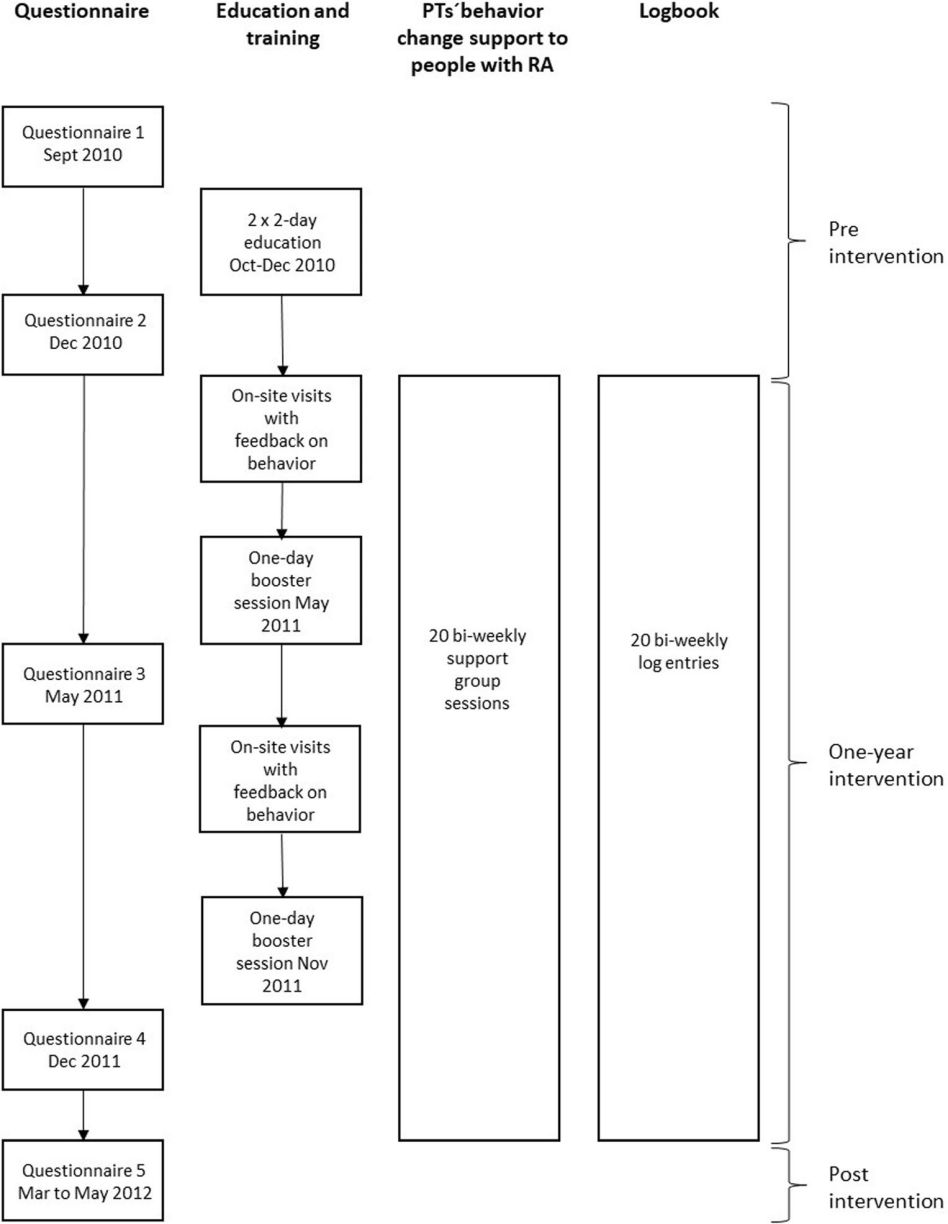
Flowchart for data collection (by questionnaire and logbook), the skills training program and the support group sessions during the HEPA intervention

Members of the PARA 2010 research team designed and provided the skills training program for PTs as outlined in Fig. 1. The program content about guiding behavior change was delivered by a trained PT with a bachelor in psychology (ID) together with a behavioral scientist/senior lecturer in psychology. The program content about HEPA recommendations and their applications in people with RA was delivered by three trained PTs with expertise in rheumatology (CO) and exercise physiology. All five teachers had long experience from teaching PTs at graduate and advanced levels in their respective fields of expertise. The PT education program was designed to increase PTs’ *knowledge* about BCTs and physical activity in RA, reduce any *fear-avoidance beliefs* related to participants’ physical activity, and increase *self-efficacy* and *skills* to guide participants to HEPA by use of specific BCTs. The BCTs to be used during support group sessions were selected based on evidence of their effectiveness in changing behavior related to physical activity [6, 7]. The PTs thus practiced how to guide HEPA intervention participants in SMART goal setting (specific, measurable, acceptable, realistic, and time-bound) [28], self-monitoring of physical activity, reviewing behavioral goals and, at the end of the HEPA intervention, coping planning to prevent relapses. Five specific PT guiding behaviors were emphasized to be performed repeatedly during the support group sessions (Table 1). The PTs also practiced how to facilitate peer support among the intervention participants.

**Table 1:** Desired behavior in HEPA participants, physical therapists’ guiding behavior and corresponding key behavior change techniques (BCTs)

Desired behavior in HEPA participants	Physical therapist guiding behavior	Key BCT
Performing physical activity according to goal setting and reflect upon revision of goals	Follow-up on goal setting from the last group meeting	Review behavioral goal(s)
Performing physical activity at high enough intensity	Follow-up on the intensity of performed physical activity	Review behavioral goal(s)
Perform physical activity and try to overcome obstacles	Provide positive feedback on performed physical activity and/or overcoming obstacles	Feedback on behavior, social reward
Perform self-monitoring of physical activity in individual journal	Provide positive feedback on self-monitoring, e.g. on the intervention participants’ journal for weekly physical activity	Feedback on behavior, social reward
Set (SMART*) goals, including physical activity planning	Guide in SMART* goal setting for physical activity for the next two weeks	Goal setting (behavior) Action planning

*SMART; S=specific, M = measurable, A = acceptable, R = realistic and T = time-bound

A 4-day course (total 24 h) including lectures and workshops was organized at two 2-day occasions prior to the HEPA intervention, along with two booster sessions (total 10 h) during the intervention. Course days and booster sessions were held in a classroom and in gym facilities at a university campus in Stockholm. The workshops were arranged so that the teachers modeled behavior guidance, e.g. guiding in SMART goal setting for physical activity, follow-up on goal-setting, giving specific feedback on performed PA and facilitation of peer support. The PTs then prepared and performed role-plays in small groups, taking the roles as PT, HEPA intervention participants or observer. The teacher modelled by using prompts like “So XX says it is hard to perform her exercise exactly according to plan. Do you have any comments to help her on the way?” Teacher-led discussions about what worked and what might be managed differently were held after the role-plays. Individual home assignments, where PTs set their own individual goals for practicing new skills related to behavior change guidance in their regular clinical practice, were performed between course occasions. Examples of home assignments were to guide a patient in setting specific behavioral goals and to provide specific feedback on patient performance. The teachers systematically focused on positive feedback to the PTs during lectures, role-plays and when the PTs reported on their home assignments.

Two individual on-site visits with video recordings were made to each PT from a research team member (ID) during the intervention year. Individual feedback was arranged so that ID asked each PT before group sessions about situations where they had felt uncertain or found it challenging to manage their guiding task, such as prompting SMART goal setting when the group participants were not motivated to set goals. ID then observed and took brief notes during the group sessions and gave positive feedback after the session on each of the five key guiding behaviors that they performed correctly, i.e. by using questions and prompts practiced during the training program. Feedback was also given on the PTs’ facilitation of peer support and how she could improve her guiding, which was typically about asking more distinct questions to the group and how to explain to the participants the reasons for planning HEPA each week.

The content of the pre-intervention course days covered information on guidelines for HEPA and its safety and benefits among people with RA. It also included information on evidence-based BCTs related to physical activity, and skills training in using them when leading support group sessions. The booster sessions in the PT training program focused mainly on feedback to the PTs on how they guided and involved the participants at support group sessions specifically with respect to goal setting, action planning and follow-up of HEPA. Selected sequences from each video-recorded support group session demonstrating how PTs guided participants in using BCTs were provided on a website available to the PTs and the research team only. The skills training program was based on SCT, using modeling, observational learning, and positive reinforcement as tools to boost the PT’s self-efficacy to perform their guiding tasks. Self-regulation, also a key concept in SCT, was addressed by formulating individual home assignments with specific goal setting at a level that was deemed realistic by each PT.

### Data Collection

Data were collected using paper questionnaires and logbooks (Fig. 1). The questionnaires were administered on five occasions over a 19-month period: once before the training program, once after the 2 × 2 course days, twice during the HEPA intervention and once within two weeks after the end of the HEPA intervention. The PTs used the structured logbook as a journal to record their guiding behavior at each support group session (*n* = 20) during the intervention year.

#### Assessment Methods

Study-specific items informed by existing questionnaires were developed by the research team, all of whom were PTs with a background in rheumatology and/or behavioral medicine, to capture relevant aspects likely to change during the training and the HEPA program. The questionnaire covered four main areas:

*Knowledge about physical activity in RA* was measured using six items based on international recommendations about the type and amounts of physical activity needed to improve and maintain health [2, 3, 29]. The items focused on physical activity in people with RA (“otherwise healthy, managing their daily activities and with well-controlled disease activity”), e.g. whether physical activity should be adjusted according to pain, whether physical activity could increase disease activity, or whether it posed risk of joint damage. The response alternatives were “true” (=1), “false” (=0) or “I do not know” (=0). The total score could range from 0 to 6, with 6 representing the best knowledge.

*Knowledge about BCTs* was measured using 16 items. The first item asked the respondent to select three methods with strong evidence base (use of goal setting, self-monitoring and social support) to guide a person intending to increase his or her physical activity (score 0–3), out of 16 listed methods. Then five items asked the respondent to name each component of SMART goal setting (score 0–5) [28]. In addition, 10 open-ended items asked respondents to define five behavior change concepts and provide examples of each one: progressive goal setting, self-monitoring, positive reinforcement, identification of barriers, and relapse prevention (score 0–10). Together, the 16 items could result in a total score ranging from 0 to 18, with 18 representing the best knowledge. Replies to open-ended items were scored according to a pre-defined template. For example, requested descriptions of “progressive goal-setting” had to include increased difficulty over time and examples provided to be specific, such as “a goal for PA could be set as walking at a certain pace for 20 minutes every day of the week. When that goal is reached, a new goal should include increased time or pace”. To avoid bias and achieve inter-rater agreement on the open-ended items, a convenience sample of 10 questionnaires (two for each of the five measurement occasions) were scored by the last author and compared to the results of the first author. After discussing the results, both authors agreed on the ratings and adjusted them accordingly.

*Fear-avoidance beliefs* related to physical activity in patients with RA were assessed using four items in the Fear-Avoidance Beliefs Questionnaire (FABQ) [30] and four items in the Tampa Scale of Kinesiophobia (TSK) [31]. All eight items were subsequently adjusted to fit the PARA 2010 context, referring to “otherwise healthy individuals with RA who manage their daily activities and with well-controlled disease activity”. The items asked questions about whether physical activity could be harmful, should be avoided when painful, and whether pain could be related to injuries for people with RA. The items were measured on a scale of 1 to 6, with 1 representing “completely disagree” and 6 representing “completely agree.” The total for the eight items yields a score from 8 to 48, with higher scores indicating stronger fear-avoidance beliefs.

*Self-efficacy to guide behavior change* was measured using nine items on beliefs about one’s own capability to guide the support group sessions in specific situations: (1) to guide in specific and (2) progressive goal setting, (3) to provide feedback on physical activity and (4) feedback on self-monitoring of physical activity, (5) to allow all participants to make themselves heard, (6) to guide in problem solving, (7) particularly when the participants got stuck in discussions on obstacles, (8) to guide in developing long-term plans for physical activity, and (9) to facilitate feedback between HEPA participants on physical activity performance. The scale was developed based on Bandura’s “Guide for constructing self-efficacy scales” [32]. The items were rated on a scale of 1 to 6, with 1 representing “not confident” and 6 “very confident”. The total for the nine items yielded a total score ranging from 9 to 54, with higher scores indicating higher self-efficacy.

Before using the questionnaire, it was tested for face validity on three clinically active PTs specialized in rheumatology, and minor revisions were made. Test-retest reliability was analyzed across two measurement occasions with 7 to 10 days between with 14 PTs in rheumatology. They were all females, distinct from the present study sample, but employed at the same rheumatology clinics. The intraclass correlation coefficient for knowledge about physical activity in RA was 0.90 (95% CI, 0.70–0.97), knowledge about BCTs 0.88 (95% CI, 0.60–0.96), fear-avoidance beliefs 0.95 (95% CI, 0.85–0.98) and self-efficacy for coaching 0.98 (95% CI, 0.91–0.99). Agreement was classified as good (≥ 0.8) [33].

*Self-reported guiding behavior* was assessed by logbooks filled in by PTs after each support group session throughout the HEPA intervention year. The logbook contained a checklist of five specific behaviors: (1) follow up on previous week’s goal setting, (2) follow up on physical activity intensity, (3) provide feedback on physical activity performance, (4) provide feedback on self-monitoring of physical activity, and (5) guide in SMART goal setting (which also includes action planning). The log-book data were used to assess PTs’ adherence to the protocol. PTs could indicate use of each of the five guiding behaviors either by having (a) posed direct questions to each individual intervention participant; e.g. “Tell me about your physical activity in relation to your goal setting the past two weeks,” or (b) initiated group activity; e.g. “Please follow up in pairs your physical activity during the past two weeks in relation to your goal setting”. Answers for each item were split for analysis into “Yes” (any use at all) or “No” (no use at all).

### Data Management and Analyses

Adherence to the protocol was operationalized according to three phases over the intervention year: phase 1 (support group sessions 1–7), phase 2 (support group sessions 8–14) and phase 3 (support group sessions 15–20). Criteria for adherence were PTs’ performing “follow-up of previous week’s goal setting” and “guidance in SMART goal setting” at support group sessions during *all three phases*. Additional criteria for *phase 1* were performing at least two of the other three guiding behaviors. For *phases 2 and 3,* at least one of the other three guiding behaviors had to be performed. However, two sessions in each phase without performing any of the specific guiding behaviors were allowed, due to introduction sessions and group sessions occasionally consisting of expert lectures or physical skills training, which were also part of the program. Based on these criteria, the PTs were then categorized as “adherers” (fulfilling all criteria), “partial adherers” (fulfilling some of the criteria) or “non-adherers” (not fulfilling any of the criteria) in each of the three phases.

Descriptive statistics are presented for both questionnaire data and logbook data as medians, means, or proportions, as appropriate. Missing data in the questionnaire’s open-ended questions about BCT knowledge were coded as incorrect answers. Pairwise deletion was employed where data were missing in the logbooks. A non-parametric Friedman’s test was used to analyze changes in questionnaire answers between measurements, and the Wilcoxon signed-rank test was used for post-hoc analyses. *P* values < .05 were considered significant. Effect sizes (*r* = Z/√N) were calculated to measure the magnitude of the differences between measurement occasions.

All data were calculated using IBM SPSS Statistics, Windows, version 22 and Microsoft Excel 2007.

### Ethics

The Stockholm Regional Ethical Review Board granted approval for the study (2010/1232–31/1 and 2011/1241–32). The PTs gave informed consent before participating in the study as did support group participants with RA involved in video recordings.

## Results

All PTs (*n* = 10) completed the questionnaires at each measurement occasion. Only the open-ended questions on knowledge about BCTs contained a few missing answers: 20 out of 500 for all PTs at all measurements.

### Knowledge, Fear-Avoidance Beliefs and Self-Efficacy for Guiding Behavior Change

**Fig. 2:**
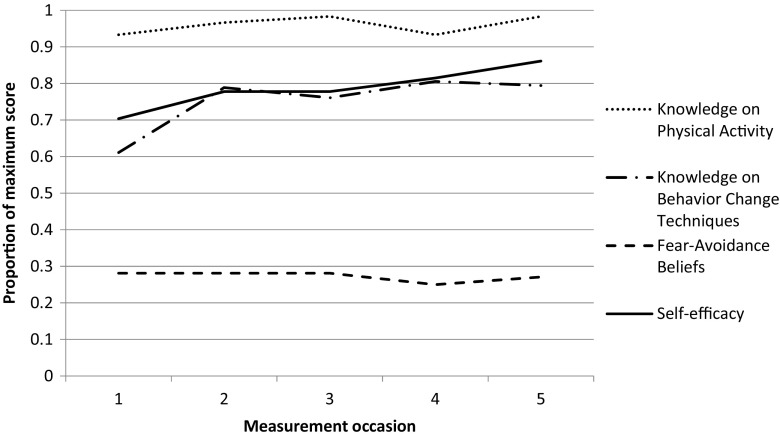
Average proportions (0–1.0) of correct answers on PTs’ knowledge about physical activity and behavior change techniques. Average median scores for fear-avoidance beliefs and self-efficacy transferred to 0–1 scores (*n* = 10)

Total scores for knowledge about physical activity and BCTs, fear-avoidance beliefs related to patients’ physical activity, and self-efficacy for guiding behavior change on the five measurement occasions are presented in Table 2 and Fig. 2.

**Table 2:** Physical therapists’ (*n* = 10) responses to a questionnaire about knowledge about physical activity in RA and behavior change techniques, fear-avoidance beliefs related to patients’ physical activity and self-efficacy to guide behavior change at five measurement occasions (M1-M5)

	M1	M2	M3	M4	M5	
	Median (Q1–Q3)	Median (Q1–Q3)	Median (Q1–Q3)	Median (Q1–Q3)	Median (Q1–Q3)	*P* ^*†*^ *(χ*^2^)
Knowledge about physical activity in RA (Score range 0–6)	6 (5–6)	6 (5.75–6)	6 (6–6)	6 (5–6)	6 (6–6)	.174 (6.4)
Knowledge about behavior change techniques (Score range 0–18)	9 (7.0–12.5)	13^*^ (11.0–13.3)	12.5^¶^ (10.0–15.0)	14.5^††^ (11.0–14.3)	13^‡^ (11.5–14.5)	.003 (16.3)
Fear-avoidance beliefs related to patients’ physical activity (Score range 8–48)	13.5 (11.8–17.5)	13.5 (10.8–15.3)	13.5 (13.0–15.5)	12.0 (9.0–13.3)	13.0 (10.0–14.0)	.119 (7.3)
Self-efficacy to guide behavior change (Score range 9–54)	38.0 (26.5–44.3)	42.0 (34.0–45.3)	42.0 (37.0–43.5)	44.0 (39.5–46.8)	46.5^§^ (43.8–51.0)	.023 (11.3)

^†^Friedmans test, *p* value significant at *p* < .05

*Wilcoxon signed-rank test M1–M2; *z* = − 2.20, *p* = .028, effect size *r* = 0.70

^¶^Wilcoxon signed-rank test M1–M3; *z* = − 1.97, *p* = .049, effect size *r* = 0.62

^† †^Wilcoxon signed-rank test M1–M4; *z* = − 2.41, *p* = .016, effect size *r* = 0.76

^‡^Wilcoxon signed-rank test M1–M5; *z* = − 2.21, *p* = .027, effect size r = 0.70

^§^Wilcoxon signed-rank test M1–M5; *z* = − 2.35, *p* = .019, effect size *r* = 0.74

PTs’ knowledge about physical activity was at a high level at all measurement occasions and did not change over time, while their knowledge about BCTs increased significantly from median 9 at the first measurement occasion to median 13 at the last. PTs’ fear-avoidance beliefs related to patients’ physical activity were low on all measurement occasions and did not change over time. PTs’ self-efficacy to guide behavior change increased significantly from median 38 at the first measurement occasion to 46.5 at the last (Table 2).

## Adherence to the Protocol on Guiding Behavior

The logbook data from the support group sessions were missing for 0 to 20% of all support group sessions, except the 1st, 16th, 17th, 19th and 20th sessions, where the rate of missing data ranged from 30 to 50%.

**Fig. 3:**
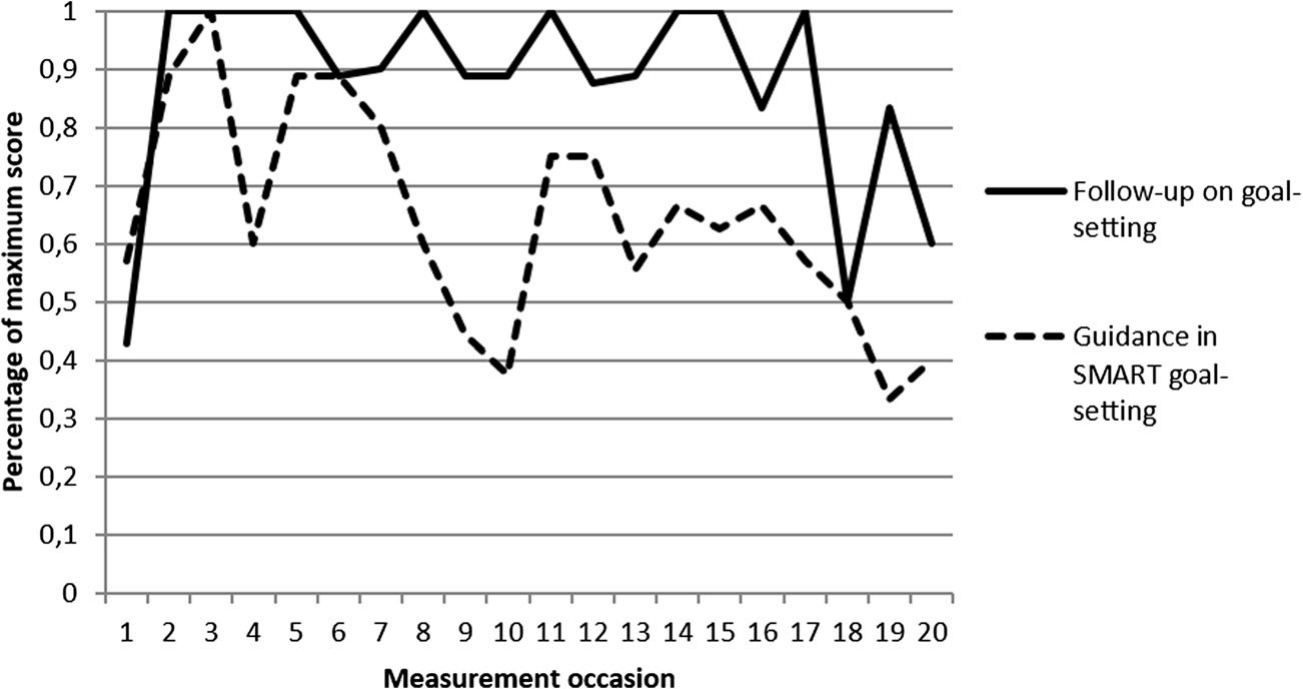
Percentage of maximum score for PTs’ self-reports of “Follow-up on previous week’s goal setting” and “Guidance in SMART goal setting” during support group sessions 1–20

Two out of ten PTs were categorized as adherers according to the pre-set criteria during all three phases. The remaining eight PTs were all categorized as partial adherers, with different patterns of adherence, partial adherence and non-adherence in each phase (Table 3). PTs’ performance of “follow-up on previous week’s goal setting” and “guidance in SMART goal setting” is presented in Fig. 3.

**Table 3:** Physical therapists (*n* = 10) categorized as adherers, partial adherers and non-adherers according to the intervention protocol for each phase and total

Physical therapist	Phase 1 (session 1–7)	Phase 2 (session 8–14)	Phase 3 (session 15–20)	Phase 1–3 (total)
1	Adherer	Partial	Adherer	Partial
2	Partial	Non-adherer	Partial	Partial
3	Non-adherer	Partial	Non-adherer	Partial
4	Partial	Partial	Partial	Partial
5	Partial	Partial	Partial	Partial
6	Partial	Non-adherer	Partial	Partial
7	Partial	Adherer	Partial	Partial
8	Adherer	Adherer	Adherer	Adherer
9	Adherer	Adherer	Adherer	Adherer
10	Non-adherer	Adherer	Non-adherer	Partial
Total number of adherers	3	4	3	2

## Discussion

To the best of our knowledge, this is the first study to describe in detail PTs’ adoption of a theory-based skills training program for guiding people with RA to HEPA. The results demonstrate that PTs’ knowledge about BCTs and their self-efficacy in guiding behavior change increased significantly during the program. The PTs’ knowledge about physical activity in RA was adequate throughout the intervention, and fear-avoidance beliefs on physical activity among people with RA were generally low from the start. However, only a minority of PTs were fully adherent to the protocol on guiding behavior change during the HEPA intervention.

The increased knowledge about BCTs indicates that the PTs learned more about certain theoretical core aspects of behavior change. This was not surprising, as it was one explicit focus of the skills training program. Overall, self-efficacy to guide behavior change was high from the start, which could also be expected in a group of PTs volunteering for a HEPA intervention program and who were both experienced and well-educated prior to enrolling in the study. However, the PTs’ increased self-efficacy over time validates previous qualitative research interviews with the same group of PTs, indicating that they perceived themselves as successively growing into the role of a guide in behavior change [10]. Since a lack of self-efficacy may be a barrier to adherence to clinical practice guidelines [34], it is encouraging that self-efficacy can be enhanced through a skills training program that includes theory and practice, with tasks of gradually increasing difficulty and feedback on PT behavior.

While our results indicate positive changes among PTs, previous studies report mixed results of educational programs for practicing PTs. Thus, increased knowledge and skills on psychosocial risk factors in back pain did not result in changed patient perceptions about PTs’ practice [11] and only minimal integration of psychosocial factors in management of low back pain [35]. The differences may be explained by differences in settings, PTs’ baseline levels and how well the education managed to target the barriers for change [36]. The PTs were well-informed about physical activity in RA before and throughout the intervention, which left little room for improvement. While other health care professionals may feel uncertain about appropriate levels of physical activity and exercise for patients with RA [37], our results confirm that PTs are meeting expectations to possess advanced expertise in this field [38].

The PTs held few fear-avoidance beliefs related to patients’ physical activity on all measurement occasions indicating that such beliefs did not represent barriers for PTs’ advice on adequate physical activity for persons with RA in the present study. This finding contradicts previous studies reporting fear-avoidance among rheumatologists [39] and PTs treating patients with back pain [14, 40]. However, our sample was self-selected and it may well be that other PTs within rheumatology hold more fear-avoidance beliefs related to their patients’ physical activity. Interestingly, despite their adequate knowledge, high self-efficacy, and low fear-avoidance beliefs, few PTs adhered fully to the protocol on guiding behavior change; they did learn to perform the specific behaviors related to key BCTs, but did not apply them in the support group sessions to the extent intended. It has previously been suggested that PTs feel insecure and lack adequate training in guiding patients in goal setting, which was a key BCT in our study [8]. However, although the PTs in our study did provide guidance in goal setting and other BCTs during the support group sessions, they chose not to do it in full accordance with the protocol. One reason for this could be a perception that goal setting and review of goals at every support group session over a whole year was unnecessary and too repetitive. Such views were expressed among the intervention participants with RA in qualitative research interviews conducted after the completion of the intervention year [41]. This influenced some of the PTs, as reported in interviews, leading them to reduce the use of these key BCTs [10].

The limited adherence to the protocol on guiding in behavior change may be interpreted in two ways: either we failed to facilitate PTs’ appropriate guiding behavior during the training program, or we failed to set adequate criteria for adherence. If we failed in designing the program, it may be because we did not emphasize enough the reason for the need of frequent use of goal setting and review of behavioral goals. We did not foresee that the repetitive use of these BCTs would be so challenging in some of the HEPA support groups as was reported in previous analyses in the PARA program [41]. However, some PTs and support groups did not experience these challenges and found stringent use of goal setting and review of goals very helpful [41]. We may also have been too strict in setting adherence criteria. The attendance at support group sessions was average 9 (SD 6.4) and positively correlated with exercise self-efficacy [26]. It is possible that, as the PTs noticed that the intervention had effect and the participants increased their physical activity [26], the PTs considered use of BCTs unnecessary and abandoned their use of BCTs according to the protocol. This might even have been adequate and future initiatives in similar contexts may benefit from a more pragmatic approach to protocol adherence.

It may be that the best combination is a PT with a solid knowledge base in both exercise physiology and behavior change, including skills to adapt support group sessions to the participants’ needs. Clearly, the pros and cons of a group format for the HEPA support program should also be considered. There are obvious advantages, such as opportunities for peer support and learning from others, but the format also reduces the chances for tailoring behavioral support to each individual’s needs and preferences. Our results also demonstrate that PTs’ increased knowledge does not automatically transfer into clinical practice, which is in line with previous implementation studies indicating that multifaceted interventions are most likely to change clinical behavior [42, 43].

One strength of the present study is the longitudinal evaluation of changes in PTs’ knowledge, beliefs and adherence when taking part in education and skills training to guide HEPA. The use of several study-specific assessment variables across multiple measurement occasions provides detailed information. One limitation is the small sample, which cannot be expected to represent the whole PT population. Our sample consisted of experienced PTs who were generally well educated in RA and guiding behavior change and all subjects had volunteered to participate in a research study involving HEPA and behavior change for people with RA. However, generalizability was not a main focus of the study. Rather, the research was designed to describe cases among this particular PT population as an example to reflect on and build upon in future initiatives. It would have been interesting to relate the adherence among the PTs to changes in physical activity among the intervention participants. However, our PT sample was too small to allow for such analyses.

Another limitation might be that the measurement properties of the study-specific questionnaire and logbook were only partly assessed. However, experienced PTs within rheumatology assessed the questionnaire for face validity, and its test-retest reliability was good. A third limitation might be that all data were collected through self-reports and may therefore contain biases [44]. In future studies, it would therefore be useful to supplement self-reports with observational data on PT behavior, where applicable [45].

One clinical implication of the findings in this study is that even with adequate knowledge and self-efficacy, PTs may need even closer monitoring and more feedback than we provided in the present training program, in order to achieve the desired changes in clinical behavior. Such methods are in line with systematic reviews in the field, recommending use of audit and feedback in multifaceted interventions to change clinical behavior in physical therapists [46] and other health professionals [42]. Further, it has been reported that successful feedback in clinical practice relies on good timing, individualization, lack of punitiveness, and customizability (i.e. flexibility in given feedback over situations and active involvement of the person receiving feedback) [47]. In a clinical context, one feasible format might include peer learning with collaboration among PT colleagues in planning, performing and giving feedback on step-by-step changes in everyday clinical practice [48].

In conclusion, these results indicate that a theory-based skills training program for PTs guiding patients with RA to HEPA may bring a significant increase in knowledge on BCTs and increased self-efficacy among experienced PTs. However, PTs’ increased knowledge did not result in full adherence to the protocol on PTs’ guiding behavior, although it should be acknowledged that the criteria for adherence may have been too strict. Keeping this in mind, PTs’ adoption of skills training programs focusing on patients’ behavior change may need closer monitoring and more feedback than provided in our study. Direct observation of PT behavior to complement PTs’ self-reports is suggested for future studies.
